# Is epinephrine during cardiac arrest associated with worse outcomes in resuscitated patients? Effect of vasopressin and catecholamines on the migration of leukocytes

**DOI:** 10.1186/s13054-015-0840-x

**Published:** 2015-03-10

**Authors:** Franz J Wiedermann, Martina Stichlberger, Wolfgang Lederer

**Affiliations:** Department of Anaesthesiology and Critical Care Medicine, Medical University of Innsbruck, Anichstrasse 35, 6020 Innsbruck, Austria

Duma and colleagues report that, in patients who achieved return of spontaneous circulation, prehospital use of epinephrine was consistently associated with a lower chance of survival, and this association showed a dose effect and persisted despite post-resuscitation interventions [1]. The accompanying editorial comment from Ewy [2] discusses the limitations and impact of this paper.

We want to point out the preliminary results of our study where we investigated the influence of vasopressin on migration and chemotaxis of human leukocytes and on oxygen free radical release of human neutrophils *in vitro*. Vasopressin stimulates the dose-dependent migration of monocytes and neutrophils. However, preincubation of leukocytes with vasopressin decreased the migratory response to other chemoattractants [3]. This is in line with the known effect of catecholamine on the migratory response of human leukocytes [4]. Procalcitonin, a marker and mediator of the immune system, has the same effect on human monocytes [5].

In our opinion, stress hormones and the mediators of the acute-phase response, like procalcitonin, are immunosuppressive by decreasing the migration of leukocytes if they are given in non-physiological doses or for a long time or, like procalcitonin, their serum levels rise substantially (the serum levels of procalcitonin rise 300- to 500-fold in the case of shock or sepsis). This could be one explanation for the lower chance of survival in the study by Duma and colleagues.
